# Update to a randomized controlled trial of lutetium-177-PSMA in Oligo-metastatic hormone-sensitive prostate cancer: the BULLSEYE trial

**DOI:** 10.1186/s13063-021-05733-4

**Published:** 2021-11-04

**Authors:** Bastiaan M. Privé, Marcel J. R. Janssen, Inge M. van Oort, Constantijn H. J. Muselaers, Marianne A. Jonker, Willemijn A. van Gemert, Michel de Groot, Harm Westdorp, Niven Mehra, J. Fred Verzijlbergen, Tom W. J. Scheenen, Patrik Zámecnik, Jelle O. Barentsz, Martin Gotthardt, Walter Noordzij, Wouter V. Vogel, Andries M. Bergman, Henk G. van der Poel, André N. Vis, Daniela E. Oprea-Lager, Winald R. Gerritsen, J. Alfred Witjes, James Nagarajah

**Affiliations:** 1 Department of Radiology and Nuclear Medicine, Radboudumc, Geert Grooteplein Zuid 10, 6525 GA Nijmegen, The Netherlands; 2 Department of Urology, Radboudumc, Nijmegen, The Netherlands; 3 Department of Health Evidence, Radboudumc, Nijmegen, The Netherlands; 4 Department of Medical Oncology, Radboudumc, Nijmegen, The Netherlands; 5 Department of Radiology and Nuclear Medicine, University of Groningen, University Medical Center Groningen, Groningen, The Netherlands; 6 Department of Radiology and Nuclear Medicine, NKI Antoni van Leeuwenhoek Hospital, Amsterdam, The Netherlands; 7 Department of Radiation Oncology, NKI Antoni van Leeuwenhoek Hospital, Amsterdam, The Netherlands; 8 Department of Medical Oncology, NKI Antoni van Leeuwenhoek Hospital, Amsterdam, The Netherlands; 9 Department of Urology, NKI Antoni van Leeuwenhoek Hospital, Amsterdam, The Netherlands; 10 Department of Urology, Amsterdam University Medical Center, Amsterdam, The Netherlands; 11 Department of Radiology and Nuclear Medicine, Amsterdam University Medical Center, Amsterdam, The Netherlands

**Keywords:** Hormone-sensitive prostate cancer, Lutetium-177-PSMA, Metastases-directed therapy, Oligometastases, Radioligand therapy, Urologic oncology

## Abstract

**Background:**

The BULLSEYE trial is a multicenter, open-label, randomized controlled trial to test the hypothesis if ^177^Lu-PSMA is an effective treatment in oligometastatic hormone-sensitive prostate cancer (oHSPC) to prolong the progression-free survival (PFS) and postpone the need for androgen deprivation therapy (ADT). The original study protocol was published in 2020. Here, we report amendments that have been made to the study protocol since the commencement of the trial.

**Changes in methods and materials:**

Two important changes were made to the original protocol: (1) the study will now use ^177^Lu-PSMA-617 instead of ^177^Lu-PSMA-I&T and (2) responding patients with residual disease on ^18^F-PSMA PET after the first two cycles are eligible to receive additional two cycles of 7.4 GBq ^177^Lu-PSMA in weeks 12 and 18, summing up to a maximum of 4 cycles if indicated. Therefore, patients receiving ^177^Lu-PSMA-617 will also receive an interim ^18^F-PSMA PET scan in week 4 after cycle 2. The title of this study was modified to; “Lutetium-177-PSMA in Oligo-metastatic Hormone Sensitive Prostate Cancer” and is now partly supported by Advanced Accelerator Applications, a Novartis Company.

**Conclusions:**

We present an update of the original study protocol prior to the completion of the study. Treatment arm patients that were included and received ^177^Lu-PSMA-I&T under the previous protocol will be replaced.

**Trial registration:**

ClinicalTrials.gov NCT04443062. First posted: June 23, 2020.

## Background

Prostate cancer is the most common non-skin cancer in males [1]. Despite surgery or external beam radiotherapy (EBRT), between 27% and 53% of patients will have a detectable prostate-specific antigen (PSA) and present with disease recurrence [2]. If there are no curative options, patients with a short PSA doubling time (e.g., <6 months) have a worse prognosis and early androgen deprivation therapy (ADT) is the treatment of choice [3–5]. While ADT delays disease progression of patients, it is associated with significant side effects and frequently impairs the quality of life [6]. Therefore, there is an increasing interest in treatments to postpone ADT while maintaining a good quality of life.

Lately, metastases-directed therapy (MDT) (e.g., EBRT) is showing promising efficacy to postpone ADT or cure selected patients with solely low-grade treatment-related side effects. Studies have reported that approximately 30–40% of patients that underwent MDT are still without ADT 5 years post-irradiation [7–10]. Particularly, patients with a limited number of metastases (≤5 metastases), the so-called oligometastatic prostate cancer, seem to benefit from MDT [7, 9, 11, 12]. Therefore, several pivotal trials are currently investigating MDT in an oligometastatic setting.

All these studies rely on imaging modalities such as positron emission tomography (PET) to detect and target these tumor metastases [13–16]. The currently favored PET tracers in prostate cancer are Gallium-68 (^68^Ga) or Fluor-18 (^18^F)-labeled prostate-specific membrane antigen (PSMA) ligands (PSMA-11, DCFPyL, or PSMA-1007). However, PSMA ligands, such as PSMA-617 & PSMA-I&T, can also be labeled with beta emitters like Lutetium-177 (^177^Lu) for radioligand therapy to deliver high local radiation doses to tumors directly [17–22].

^177^Lu-labeled PSMA is a promising new therapeutic approach with the pending registry in the 3rd or 4th line castration-resistant prostate cancer [21]. Recently, we showed that ^177^Lu-PSMA is also highly effective in the hormone-sensitive setting with low volume disease because of high tumor uptake of PSMA targeted radioligands in small lesions, such as oligometastatic prostate cancer [17, 18, 23]. Moreover, the favorable toxicity profile of ^177^Lu-PSMA seen in our pilot study supports this new treatment in this setting. Therefore, we initiated a prospective randomized multicenter phase II study that evaluates the efficacy of ^177^Lu-PSMA in oHSPC to postpone disease progression and to avert the need for ADT. This study protocol was published in 2020, but was recently amended. The changes to the protocol are described in the present report [24].

### Changes to the protocol

The original protocol was designed using the PSMA ligand PSMA-I&T, which can be labeled with ^177^Lu in our local laboratory following GMP conditions. Unfortunately, due to COVID-19-related issues (e.g., personnel shortage, remote working, and increased demand for hospital resources), the production of ^177^Lu-PSMA-I&T for our study did not receive a priority designation in our hospitals. Therefore, the study required a third party for the stable production of ^177^Lu-PSMA and a logistical party to supply all participating centers. The study will now use ^177^Lu-PSMA-617 as Advanced Accelerator Applications stepped in for support. The study also received a new title “Lutetium-177-PSMA in Oligo-metastatic Hormone Sensitive Prostate Cancer.”

As some patients may have a residual disease with good PSMA uptake after two cycles of 7.4 GBq ^177^Lu-PSMA, with no or only low-grade side effects, the present protocol enables an additional two cycles if this is thought beneficial for patients. Thus, patients in the therapeutic arm (or those in the control arm that met the primary endpoint and now receive ^177^Lu-PSMA) are eligible for another two cycles of 7.4GBq of ^177^Lu-PSMA given 12 and 18 weeks after cycle one. To evaluate if the residual disease is present after the first two cycles, patients will also undergo an interim ^18^F-PSMA PET scan 4 weeks (±1 week) after cycle two.

### Study progress

Since recruitment began in July 2020, only three patients were included prior to the amendments reported above. One patient was allocated to the therapeutic arm and received two cycles of ^177^Lu-PSMA-I&T. This patient will be replaced by a new patient under the amended protocol. The two control arm patients will not be replaced and will be eligible to receive ^177^Lu-PSMA-617 in case they meet the primary endpoint, which is disease progression within 24 weeks post cycle two.

## Conclusion

After the publication of the original protocol, the study was amended due to COVID-19-related issues to acquire third-party support for the labeling of the radiopharmaceutical. Therefore, the study will now use ^177^Lu-PSMA-617 instead of ^177^Lu-PSMA-I&T. In addition, patients with residual disease on ^18^F-PSMA PET after the first two cycles are eligible to receive an additional two cycles of 7.4 GBq ^177^Lu-PSMA in week 12 and week 18 after cycle one, summing up to a maximum of 4 cycles if necessary.

## Data Availability

The datasets used and/or analyzed during the current study are available from the corresponding author on reasonable request.

## Abbreviations

^68^Ga: Gallium-68
^18^F: Fluor-18
^177^Lu: Lutetium-177
ADT: Androgen deprivation therapy
EBRT: External beam radiotherapy
MDT: Metastases directed therapy
oHSPC: Oligometastatic hormone-sensitive prostate cancer
PET: Positron emission tomography
PSA: Prostate-specific antigen
PSMA: Prostate-specific membrane antigen
